# Exit Interviews Use in Integrated Clinical Academic Training Programmes: An Online Survey to Elicit Current Practice in England and Wales

**DOI:** 10.1177/23821205261476109

**Published:** 2026-08-04

**Authors:** Daniel Darbyshire, Hanan Basheer

**Affiliations:** 1 Lancaster Medical School, Lancaster University, Lancaster, UK; 2 Emergency Department, Manchester Royal Infirmary, Manchester, UK

**Keywords:** medical education, clinical academic training, exit interviews, health professions education, trainee evaluation

## Abstract

**Background:**

Exit interviews are widely used in human resources practice to understand employee experiences and inform organisational improvement. In academic medicine, trainees frequently transition between clinical and academic roles during integrated clinical academic training pathways, including Academic Clinical Fellowships (ACFs) and Academic Clinical Lectureships (ACLs). However, the use of exit interviews in this context is not well described.

**Objective:**

To explore the use of exit interviews within integrated clinical academic training (ICAT) programmes in England and Wales, and to identify existing practices, themes, and alternative approaches to trainee feedback.

**Methods:**

An online survey was distributed to ICAT leads across England and Wales via the InterACt network in May 2025. The survey addressed whether exit interviews were offered, their structure and content, and other feedback mechanisms. Responses were collected via email, collated using Microsoft Forms, and analysed descriptively.

**Results:**

Twenty responses were received. Only 4/20 (20%) programmes reported offering exit interviews for both ACFs and ACLs, while half (10/20) had no formal process. Practices ranged from structured, mandatory interviews to informal discussions. Where conducted, some programmes used templates or checklists, while others employed semi-structured approaches. Common themes included challenges in balancing clinical and academic responsibilities, delays in initiating research, and uncertainty around clinical academic career pathways. For ACLs, lack of bridging funding and limited post-CCT opportunities were key concerns. Positive experiences included strong institutional support, networking opportunities, and the value of induction and interim reviews. Most programmes (13/20) used alternative methods to collect feedback, particularly surveys (annual, exit, and alumni), alongside ARCP discussions and informal meetings.

**Conclusions:**

Exit interviews are not routinely implemented across ICAT programmes, although interest in their adoption is evident. Practices vary considerably, with surveys commonly used as an alternative feedback mechanism. Findings support development of a standardised exit interview approach tailored to clinical academic training.

## Introduction

Medical research and the clinical academic workforce are vital for the health and wealth of the UK. However, the number of clinical academics has been steadily declining, with a significant drop in those in senior positions. This field has an ageing workforce, with a large proportion of clinical academics nearing retirement age, leaving a gap in the pipeline for new researchers and educators. Factors such as heavy workloads, precarious contracts, and below-inflation pay are suggested as leading many academics to leave the field.^1,2^

A 2021 publication reported the results of a thematic analysis combining an online survey with semi-structured interviews among clinical academic trainees in the East Midlands. The authors identified challenges in balancing and progressing both the clinical and educational aspects of participants' careers. Clinical academic trainees developed self-efficacy with the support of peers, supervisors, and mentors. The authors suggested that further work was needed to streamline the clinical academic training pathway and increase diversity.^3^

Despite attempts to address the problems, clinical-academic training, like academic medicine more broadly, remains, as the editor of the BMJ puts it, “broken.”^4^ One barrier to improvement is the constantly changing landscape of postgraduate medical training. A second barrier is the lack of information about what works and does not work in integrated clinical-academic training.

The United Kingdom’s National Health Service (NHS) has conducted an electronic exit questionnaire for staff leaving the NHS. A small number of trusts have successfully implemented exit interviews to improve staff well-being and retention.^5^ However, exit interviews are far from commonplace, and their limited implementation is a lost opportunity to learn about our workforce, workplaces, and training. Significant barriers to conducting exit interviews include the time required and the power gradient that often exists between the interviewer and interviewee.

In academic medicine, trainees frequently move between clinical and academic roles—for example, during Academic Clinical Fellowships (ACFs) and Academic Clinical Lectureships (ACLs)—which require balancing clinical training with research activity and are associated with recognised challenges. Exit interviews may therefore provide a mechanism to better understand trainee experiences within integrated clinical academic pathways and to inform programme improvement.^6^

Conducting exit interviews is an established Human Resources practice when individuals leave roles and is widely used to understand employee experience and organisational performance. Exit interviews are commonly viewed as a tool to identify causes of turnover and inform improvements in organisational processes, enabling feedback from departing staff to refine systems and practices. While traditionally associated with employees leaving organisations, this approach may also be valuable in other contexts involving role transition.^7,8^

To start developing a programme of research and improvement using exit interviews, it is important to understand how widespread their use is and how they are used in places already implementing them. Addressing this gap in the literature was the primary aim of this study.

## Methods

To understand this, we conducted an online survey, which was sent out to all Integrated Clinical Academic Training (ICAT) leads in England and Wales via the InterACT group on the 21st March 2025, with the final response collected on the 8^th^ April 2025. Each university delivering ICAT has an ICAT lead, usually a senior clinical-academic. We did not capture the respondents' demographic details. The survey asked three questions:

1. In your region, are exit interviews undertaken/offered to ACFs and/or CLs?
2. If yes, do you follow a particular structure? Are there any themes from the interviews that you can share?
3. Do you collect information from ACFs or CLs in any other way?

The questions were developed by consulting key methodological texts^9^ and early work from an ongoing scoping review on the use of exit interviews in postgraduate medical education.^10^

The answers were returned via email, collated in Microsoft Forms, and exported to Microsoft Word for review. We received 20 responses.

## Results

### Q1: In Your Region, are Exit Interviews Undertaken/Offered to ACFs and/or CLs?

**Figure 1.**
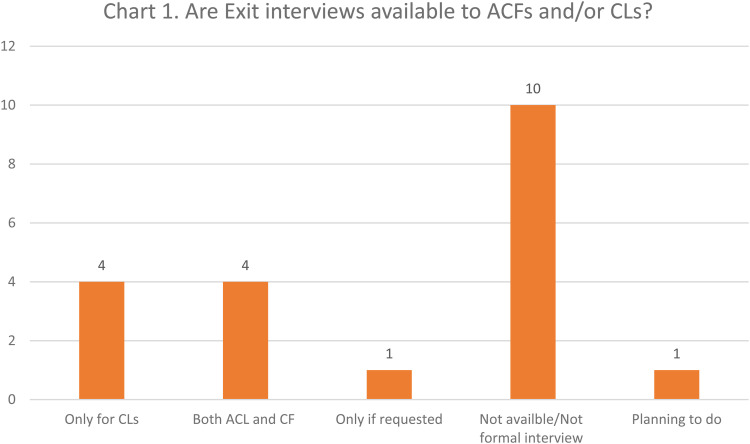
Bar graph representing the responses received for question 1.

Most programmes do not offer exit interviews, with only four of the twenty responses (20%) offering them to both ACFs and ACLs. Of those who do not offer them, at least one is because the programme is in its infancy and has not had ICAT resident doctors complete their leave. Figure 1 summarises exit interview availability.

Some institutions provide formal, structured meetings for both roles to gather feedback and discuss future career paths; others offer them on an informal, request-based basis. Several programmes currently lack a standardised process but express significant interest in implementing formal systems to better support trainee development and programme improvement. In contrast, certain locations use annual progress reviews or departmental catchups as a substitute for a final interview. Ultimately, the data reflect a diverse landscape, ranging from robust, mandatory exit protocols to a complete absence of formal feedback mechanisms during the transition out of academic training. available.

Full responses to this question are available in Appendix 1.

### Q2a. If Yes, Do You Follow a Particular Structure?

Three programmes used a structure to guide the exit interview, with one programme asking participants to complete a document in advance. Two programmes provided the documents used to support the exit interviews. One of which is a checklist of the various Human Resources processes that occur on leaving an organisation, such as returning IT equipment, ensuring adequate data stewardship for research data, and transition arrangements for services such as email access. The second is more ACL-focused, with questions around achievements such as publications and grant income, plans for fellowships, and how the ACL has found the programme and how it might improve.

**Figure 2.**
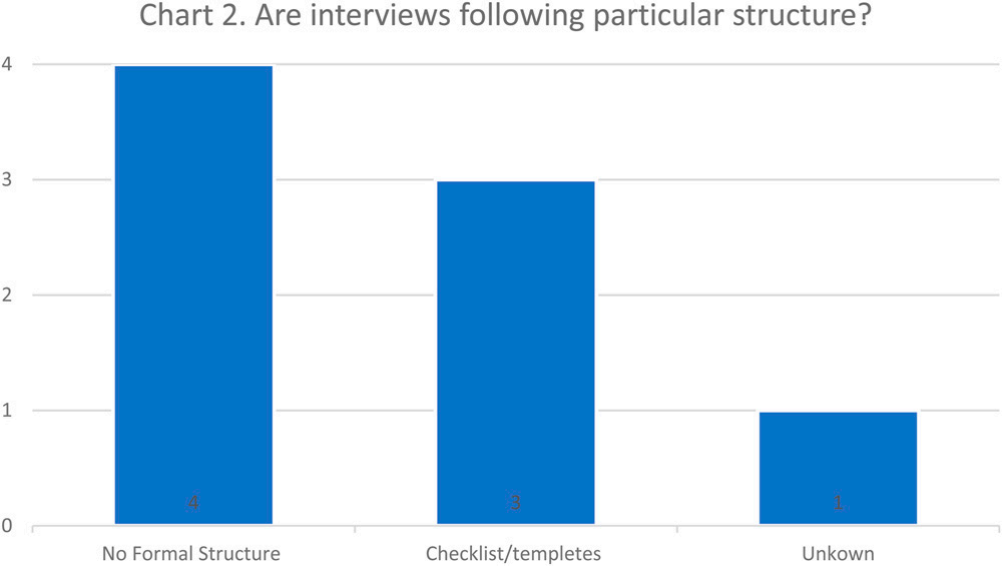
Bar graph representing the responses received for question 2.

The four responses that did not follow a particular structure, however, covered similar areas. Common questions included what worked well in the programme, what could be improved, and what the person plans after this role. Figure 2 summarises exit interview structure.

Complete responses are available in Appendix 2.

### Q2b: Are There Any Themes From the Interviews That You can Share?

Common themes can be divided into challenges and positives: Challenges include the difficulties of balancing clinical and academic roles, delays in starting initial projects, particularly for ACFs, and uncertainties about the clinical academic career pathway in general and difficulties in getting PhD positions. Particularly for ACLs, the lack of bridging funds and permanent career posts for post-ACL, post-CCT work is a concern.

Positives include valuing networking opportunities, some reports of positive support from the local University clinical academic training team, the utility of interim reviews, and the value of induction. The importance of the speciality TPD for the academic trainees’ experience is also noted in one response, as is a desire for more opportunities for ICAT resident doctors to meet informally/socially.

Complete responses are available in Appendix 2.

### Q3. Do You Collect Information From ACFs or CLs in Any Other Way?

**Figure 3.**
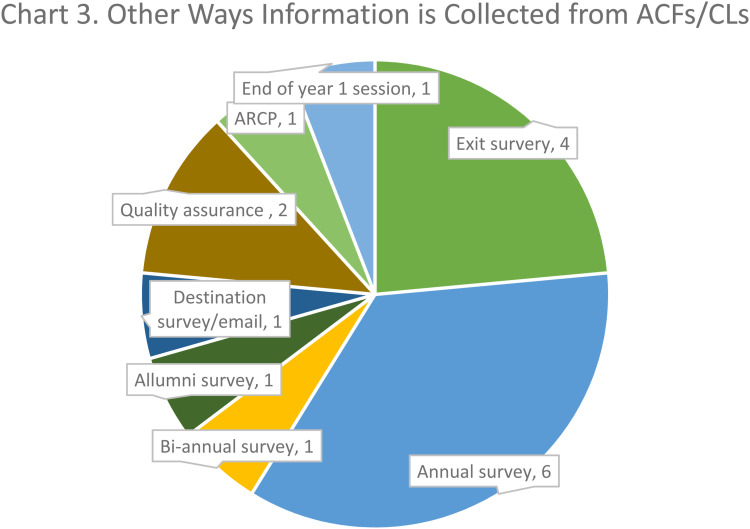
Pie chart representing the responses received for question 3.

Thirteen respondents provided information on different ways they collected information from ACFs and ACLs. Nine respondents reported using surveys to gather information from their ACFs and/or ACLs: seven conducted annual surveys, one conducted a bi-annual survey, four conducted exit surveys, and one conducted a destination survey/email. Some programmes used multiple surveys. One respondent utilised the ARCP paperwork and an informal catch-up with the ATPD, and a further two utilised a specified quality assurance framework involving annual meetings between the trainee and those leading the programme. One programme had a dedicated end-of-year 1 session. Figure 3 summarises other ways information is collected from ACFs/CLs.

## Discussion

This study demonstrates that exit interviews are not routinely implemented across ICAT programmes, with most relying instead on surveys or informal discussions. This lack of standardisation reflects a broader limitation in clinical academic training: insufficient systematic data on trainee experience to inform programme development.

The challenges identified—particularly balancing clinical and academic commitments, uncertainty around career progression, and limited post-training opportunities—are consistent with previous studies of ICAT pathways. At the same time, reported positives such as strong institutional support and networking opportunities mirror existing literature, reinforcing the importance of capturing both strengths and areas for improvement at key transition points.^6^

Exit interviews offer a structured mechanism to achieve this. Within human resource practice, they are widely used to identify organisational strengths and weaknesses, understand reasons for leaving, and inform quality improvement. Common themes typically include career development, workplace environment, supervision, and organisational culture—domains that closely align with those identified in this study. This suggests that established exit interview frameworks could be readily adapted for ICATs.^8^

Barriers to implementation, including time constraints and potential power imbalances, are well recognised. However, these may be mitigated through semi-structured approaches, use of independent interviewers, and integration with existing feedback systems. Importantly, exit interviews provide a distinct opportunity to capture reflective, end-of-programme insights not accessible through routine surveys.

In the context of ongoing concerns regarding the clinical academic workforce, introducing structured exit interviews represents a pragmatic and potentially high-yield strategy to support evaluation and improvement of ICAT programmes.

## Supplemental Material

Supplemental Material - Exit Interviews Use in Integrated Clinical Academic Training Programmes: An Online Survey to Elicit Current Practice in England and Wales Supplemental Material for Exit Interviews Use in Integrated Clinical Academic Training Programmes: An Online Survey to Elicit Current Practice in England and Wales by Daniel Darbyshire, Hanan Basheer in Journal of Medical Education and Curricular Development

## Data Availability

All the data for this article is included in Appendices 1 to 3.*
