# Supplementary material for: Exit Interviews Use in Integrated Clinical Academic Training Programmes: An Online Survey to Elicit Current Practice in England and Wales
Source: J Med Educ Curric Dev. 2026 Aug 4;13:23821205261476109. doi: 10.1177/23821205261476109 (PMC13438350; doi:10.1177/23821205261476109)
Supplement: Supplementary file 1 [file sj-pdf-1-mde-10.1177_23821205261476109.pdf]

Appendix 1. Responses to Question 1: In your region, are exit interviews undertaken/offered to ACFs and/or CLs?

Yes, both ACLs and ACFs

1. No idea about the region, but yes for Imperial-linked ACFs and CLs – all.
2. We do - these are offered to everyone, and we get really high uptake (usually only one person doesn't, and often because of diary challenges). We also aim for annual catch-up meetings - all directly with the ATPD.
3. Yes, we do these by the CAT team, not trainees, as reasons for drop out are often medical or health-related, so confidentiality applies.
4. We have an exit "discussion" where we collect feedback about the training scheme but importantly discuss their future careers and opportunities.

Yes, only ACLs

1. Currently, only CLs, but we are keen to develop a more formal process for the ACFs as well, so we are keen to hear more and are supportive of your project.
2. We have an annual academic ARCP for ACF and ACL. CLs leaving the university should also be offered an exit interview by their line manager
3. CLs are offered an exit interview with the ACL lead upon completion. We do not currently have an exit interview for ACFs but are looking to implement this soon.
4. In East Midlands South (Leicester), we hold an exit interview with all our ACLs, but not ACFs. This is on Teams these days, with 2 members of the clinical academic training operation group (including the director) present. The things we cover during these include general feedback on their ACL experience, both positives and any challenges encountered, their next destination, any future plans to remain in Academia/Fellowships (if not already obtained) and any suggestions for improving the programme at Leicester.

Yes, upon request

1. We currently do not offer this as standard unless the trainee requests an exit interview.

No

1. Not formally, but quite often, I will catch up with trainees before they finish with us to check on their next steps and how they can be supported, and often ask for informal feedback. I would actually like to formalise this process as I think it would be helpful.
2. We don’t do exit interviews, but it sounds interesting.
3. We do not have a formal system for exit interviews, but through the NHRP Academy community, we have an ongoing commitment to support ACFs and CLs with their academic career development, e.g. applications for PhD or Intermediate Fellowships, a rolling series of events, etc. We do meet with anyone who has any specific problems or questions that need addressing as they finish the programme.

4. The Cambridge training scheme does not do exit interviews, but I would be interested to hear of others' experiences.
5. As a new medical school (Sunderland), our first cohort of ACFs started in 2024, and so we are a couple of years away from exit interviews, etc.
6. Not to ACFs, I understand. May be offered to some ACLs, as they are University employees, but I don't think that this is mandated
7. We don't have exit interviews in Wales
8. We don't offer exit interviews to our ACFs and CLs as standard, although I do try to meet with all of our IATs on a regular basis.
9. I'd be keen to explore undertaking structured exit interviews as I suspect these would be hugely valuable.
10. In Newcastle, in conjunction with NHRP Academy and NHSE Education North East, we meet each year with each ACF and CL, with IAT Lead and Deputy Lead, Academic Supervisor and Deputy PostGrad Dean - these meetings are designed to be constructive and relaxed with the intention to deliver individualised advice and to act on any issues promptly, e.g. clinical placements, academic time being protected, etc

Plan to do exit interviews

1. We are just establishing a portfolio of ACFs, and I hope ACLs. It will be part of our then-established management process to ensure that the academic Surgical Unit at RPH gathers feedback from exit interviews of all our future academic staff. Note that we are very pleased to announce that we have 1 ACF and hope to boast a second in 2026 with our first ACL in 2025 and second in 2027.

Appendix 2. Responses to Question 2: If yes, do you follow a particular structure? Are there any themes from the interviews that you can share?

If yes, do you follow a particular structure?

Yes, follow a checklist or template

1. Yes – CLs are treated like all UoB employees, and it is mandatory that line managers (usually a tenured clinical academic at SL or above level does this. There is a checklist of issues that need to be discussed (attachment provided)
2. We hold our academic ARCP against a review template.
3. In advance of the CL exit interview, they prepare the exit interview document, which is then discussed at this meeting (attachment provided)

No particular structure

1. Generally, just an informal chat asking what we could do better, what was helpful etc.
2. No formal structure but collect information on dept and university and clinical TPD support, progress, problems and successes, next steps, etc... We produce annual reports about this and feedback widely.
3. There is no "format" as it is tailored to the individual.
4. Semi-structured: How are you? What are you doing now/will be doing/want to be doing? What bits of the IAT could have done better for you? What are the best bits that we should fight to keep going?

Unknown

1. The discussions would be with the ACL's line manager and would not be shared with us at ICAT

Are there any themes from the interviews that you can share?

1. I think general themes would include: the challenges of balancing the roles, delays in getting started with an initial project (for ACFs) and having some 'false starts', some uncertainties around the pathway as a whole (especially for CLs).
2. Positives – support offered by University CAT team, fast responses, offer of interim reviews, opportunities for networking between training levels welcomed, induction & summer meeting important, would like more opportunities to meet informally/socially.
3. Some themes from recent ACL exit interviews are as follows:-
   Positives – support offered by University CAT team, fast responses, offer of interim reviews, opportunities for networking between training levels welcomed, induction & summer meeting important, would like more opportunities to meet informally/socially.
   Negatives – challenges sometimes with clinical/academic balance, particularly in small specialities where TPD does not have a full understanding of supernumerary posts; Lack of interim bridging opportunities for ongoing clinical-academic posts after the end of ACL and prior to Advanced Fellowships/CCT, to allow momentum to continue.
4. For ACFs, the recurring thing is the difficulty in getting PhD positions and the lack of a BRC in Norfolk, which means they do not have soft research money for one year prior to applying for

PhD fellowships. For CLs (and in all fairness, we only had two completing in the last two years- it is the difficult research prospects and minimal advertised posts...

Appendix 3. Responses to Question 3: Do you collect information from ACFs or CLs in any other way?

Surveys

1. Yes. Annual surveys of all, 1:1 sessions at the end of year 1 for ACFs and CLs, drop-in sessions for all (poorly attended) open access CATO office ...
2. However, we do ask leaving trainees to complete a short survey, in which we ask them what their next destination will be, and a few questions about their experience. (Survey link provided to author).
3. Yes – we send an annual census to ACFs and CLs. Additionally, we send a leaver survey when they complete their programmes. We periodically send an update survey to alumni to determine whether the trainees remain in clinical academia or are pursuing a career in research.
4. Here we run an ACF and CL Leavers Survey every year, and this captures trainees' experiences of our programme as well as destinations, if known. It's not compulsory but we build in reminders and get a decent response rate.
5. We have a yearly feedback survey for all ACFs and CLs, and we also contact them after they have left to collect destination data.
6. Each year we gather feedback from ACFs and CLs out with the progress meetings, etc, via online form/survey.
7. We gather information about the destination via academic reviews and ad hoc when trainees are leaving, for information we need for NIHR reporting or the bid. We also do an annual feedback survey.
8. In addition, we have one annual survey (electronic and anonymous) every year where we collect feedback, undertaken using Microsoft Forms.
9. A biannual trainee experience survey, last run in Jan 2025.

Other means

1. The structure of our QA process/support for trainees aligns with that of Newcastle (IAT leads copied in) and happens regionally via our Deanery.
2. Every year we ask people to send us their academic paperwork they do for the ARCP and have a 'catch up' chat with the ATPD or Deputy for chances for niggles to be shared, so we can share in the things they want to boast about and pick up annoying issues.
3. Yes, for ACFs, we will email them in advance of completion, asking them for information on their next destination. We also will check with the academic programme leads on progress of ACF/CLs in their speciality.
4. In Newcastle, in conjunction with NHRP Academy and NHSE Education North East, we meet each year with each ACF and CL, with IAT Lead and Deputy Lead, Academic Supervisor and Deputy PostGrad Dean - these meetings are designed to be constructive and relaxed with the intention to deliver individualised advice and to act on any issues promptly, e.g. clinical placements, academic time being protected, etc.

Appendix 4. Invitation Email to Complete Exit Interviews Survey

Dear all,
Please see below a message from our InterACt Trainee Representatives, for all IAT Programme Leads.

Dear IAT Leads,
I have been working with my fellow InterACt ACF and CL reps on a project idea to develop peer led exit interviews with those finishing an academic training programme. I understand from Professor Barratt that some regions may already be doing this, so before taking this idea forward it would be useful to know the answers to the following questions:

1. In your region are exit interviews undertaken/offered to ACFs and/or CLs?
2. If yes, do you follow a particular structure? Are there any themes from the interviews that you can share?
3. Do you collect information from ACFs or CLs in any other way?

Please respond directly to myself at d.darbyshire@lancaster.ac.uk with your responses.
Many thanks for any information or thoughts you are willing to share with myself and the other InterACt Trainee Representatives.
Sincerely,
Dan

Dr Daniel Darbyshire (He/Him)
NIHR Academic Clinical Lecturer, Lancaster Medical School
ACL Representative, Integrated Academic Clinical Training (InterACt) Committee

---

Best Wishes,
Charlotte
(InterACt Coordinator)
